# Supplementary material for: An acute microglial metabolic response controls metabolism and improves memory
Source: bioRxiv. 2024 Apr 25:2023.04.03.535373. Originally published 2023 Apr 4. Preprint. [Version 2] doi: 10.1101/2023.04.03.535373 (PMC10103996; doi:10.1101/2023.04.03.535373)
Supplement: Supplement 1 [file NIHPP2023.04.03.535373v2-supplement-1.pdf]

bioRxiv preprint doi: https://doi.org/10.1101/2023.04.03.535373; this version posted April 25, 2024. The copyright holder for this preprint (which was not certified by peer review) is the author/funder, who has granted bioRxiv a license to display the preprint in perpetuity. It is made available under aCC-BY 4.0 International license.

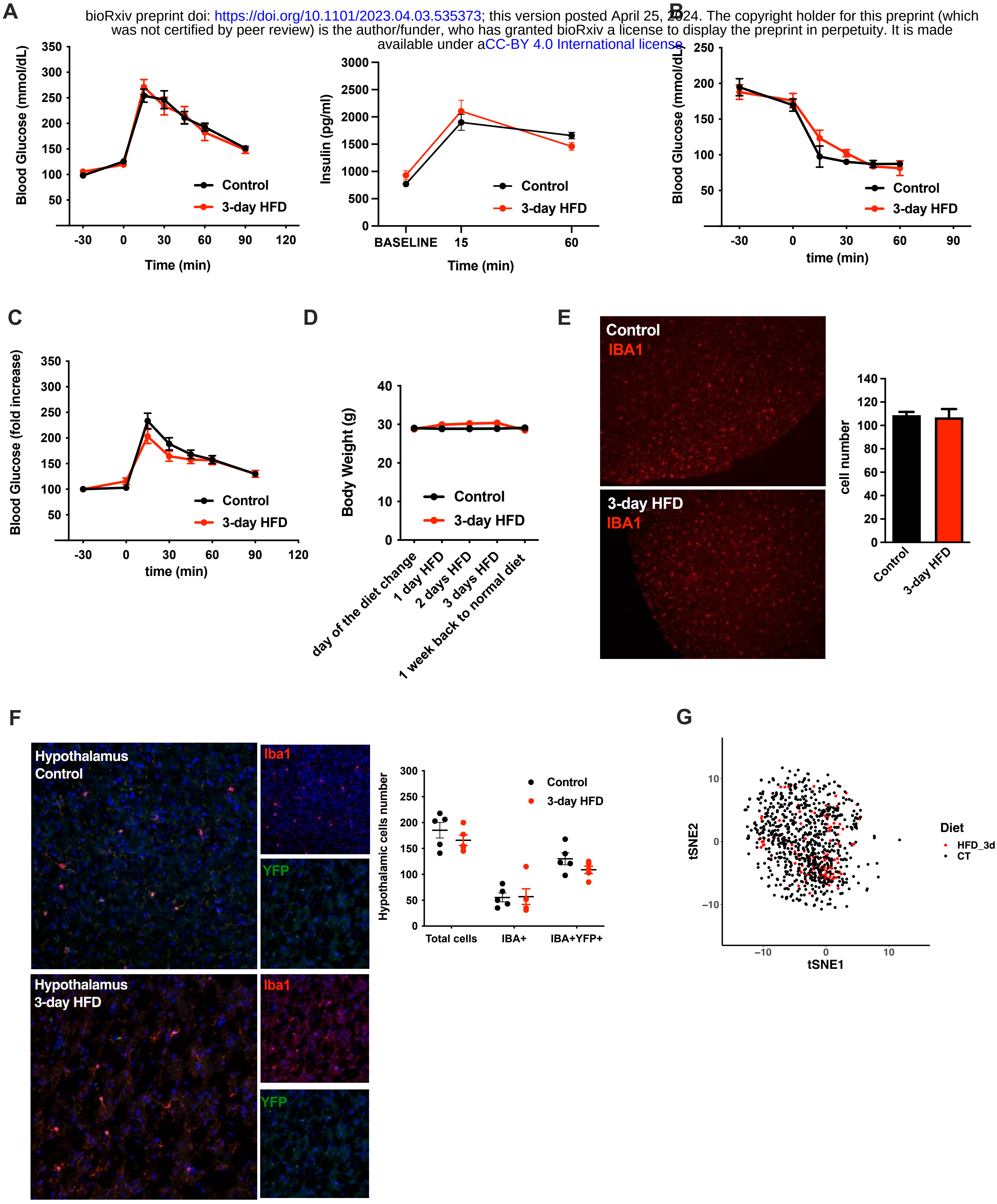

Supp_Figure_1

bioRxiv preprint doi: https://doi.org/10.1101/2023.04.03.535373; this version posted April 25, 2024. The copyright holder for this preprint (which was not certified by peer review) is the author/funder, who has granted bioRxiv a license to display the preprint in perpetuity. It is made available under aCC-BY 4.0 International license.

Metabolite	*FC*	*log2FC*	*P.value*	*log10.P.Value*	*Enrichment*
KynurenicAcid	3.56	1.83	0.24	0.62	Not Sig
HexadecanoicAcid	2.27	1.18	0.01	2.11	HFD 3d
Nicotinamide-N-oxide	0.45	-1.14	0.02	1.71	CT
OctadecanoicAcid	2.17	1.12	0.02	1.64	HFD 3d
Serotonin	0.51	-0.98	0.95	0.02	Not Sig
N-Methyl-4-Pyridone-3-Carboxamide	1.81	0.85	0.06	1.21	Not Sig
Methylmalonate	1.80	0.85	0.01	1.86	Not Sig
TetradecanoicAcid	1.78	0.83	0.29	0.53	Not Sig
Octanoylcarnitine	0.60	-0.73	0.99	0.00	Not Sig
Propionylcarnitine	1.65	0.73	0.45	0.35	Not Sig
Isovalerylcarnitine	1.65	0.72	0.02	1.74	Not Sig
NicotinicAcid	1.60	0.68	0.04	1.39	Not Sig
Glutarylcarnitine	1.60	0.68	0.00	2.40	Not Sig
Nicotinamide	0.63	-0.66	0.07	1.13	Not Sig
1-methylnicotinamide	0.66	-0.59	0.31	0.51	Not Sig
OphthalmicAcid	0.68	-0.56	0.24	0.62	Not Sig
N-methylserotonin	1.41	0.49	0.17	0.77	Not Sig
O-Acetylcarnitine	1.38	0.47	0.62	0.21	Not Sig
Tryptophan	1.35	0.43	0.10	1.02	Not Sig
3-hydroxyanthranillicAcid	1.34	0.43	0.20	0.69	Not Sig
Butyrylcarnitine	1.33	0.42	0.28	0.55	Not Sig
Nudifloramide	1.27	0.34	0.04	1.41	Not Sig
QuinolinicAcid	1.21	0.27	0.22	0.66	Not Sig
S-Adenosyl-L-Homocysteine	0.84	-0.26	0.44	0.35	Not Sig
Isobutyrylcarnitine	1.15	0.21	0.62	0.20	Not Sig
2-MethylbutyrylCarnitine	1.15	0.20	0.64	0.19	Not Sig
NicotinicAcidMononucleotide	0.90	-0.15	0.33	0.49	Not Sig
Hexanoylcarnitine	1.10	0.14	0.69	0.16	Not Sig
NicotinamideMononucleotide	1.10	0.14	0.95	0.02	Not Sig
Carnitine	1.06	0.08	0.88	0.06	Not Sig
S-Adenosyl-L-Methionine	1.06	0.08	0.61	0.21	Not Sig
3-Hyroxykynurenine	1.04	0.06	0.87	0.06	Not Sig
NicotinuricAcid	0.96	-0.06	0.64	0.19	Not Sig
AnthranillicAcid	1.04	0.05	0.96	0.02	Not Sig

TABLE_1

was not certified by peer review) is the author/funder, who has granted bioRxiv a license to display the preprint in perpetuity. It is made available under aCC-BY 4.0 International license.

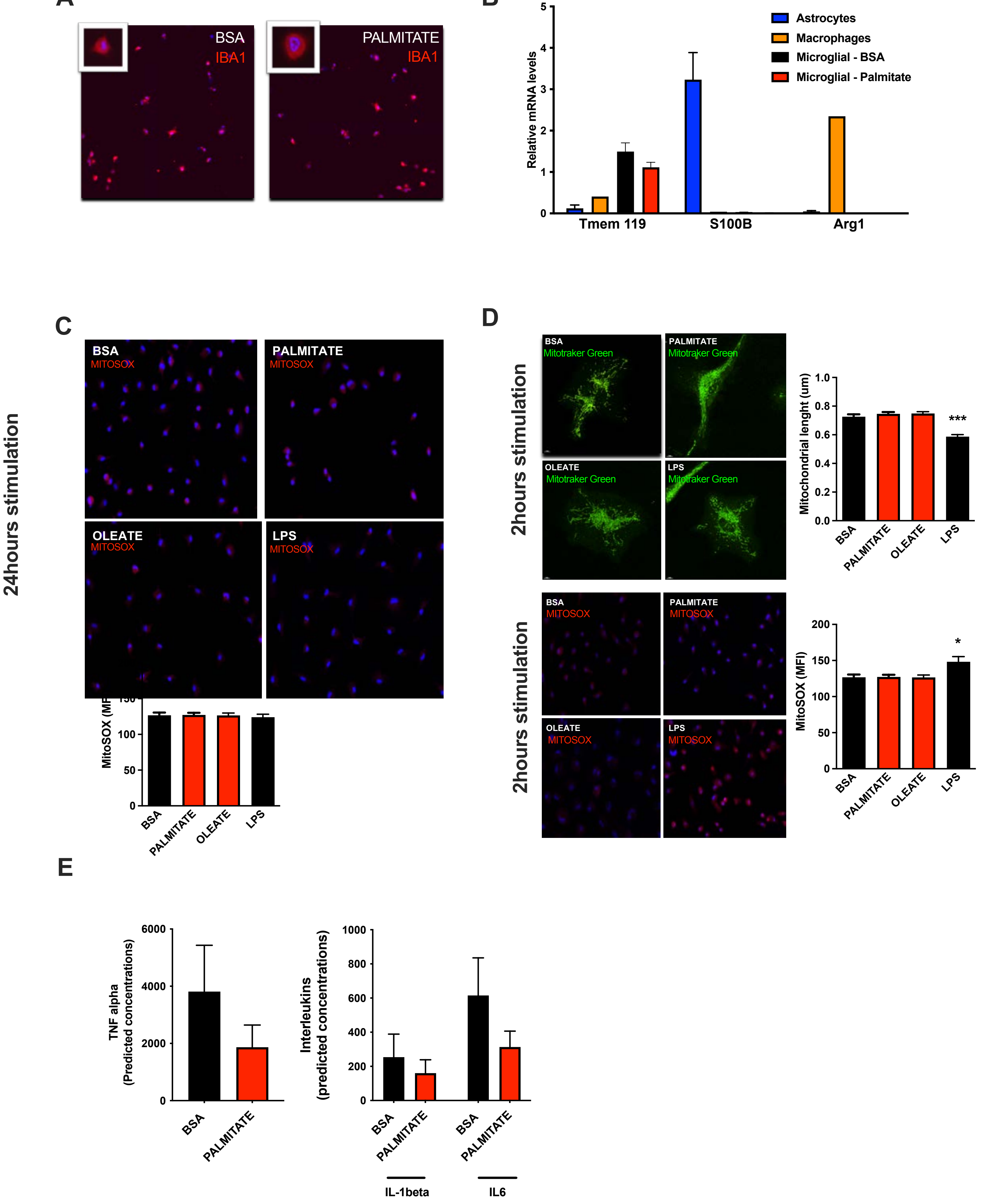

Supp_Figure_2

was not certified by peer review) is the author/funder, who has granted bioRxiv a license to display the preprint in perpetuity. It is made available under aCC-BY 4.0 International license.

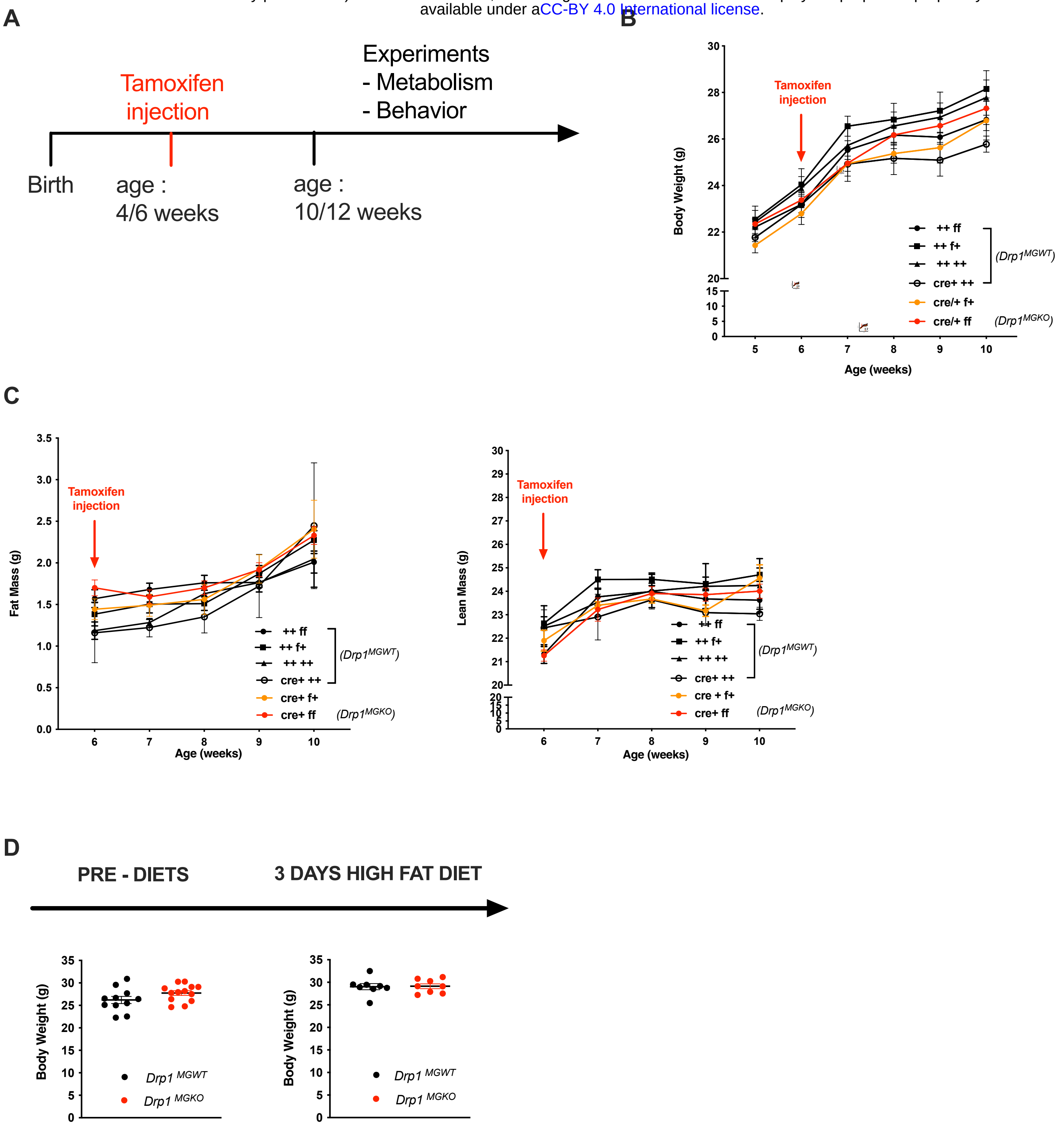

Supp_Figure_3

bioRxiv preprint doi: https://doi.org/10.1101/2023.04.03.535373; this version posted April 25, 2024. The copyright holder for this preprint (which was not certified by peer review) is the author/funder, who has granted bioRxiv a license to display the preprint in perpetuity. It is made available under aCC-BY 4.0 International license.

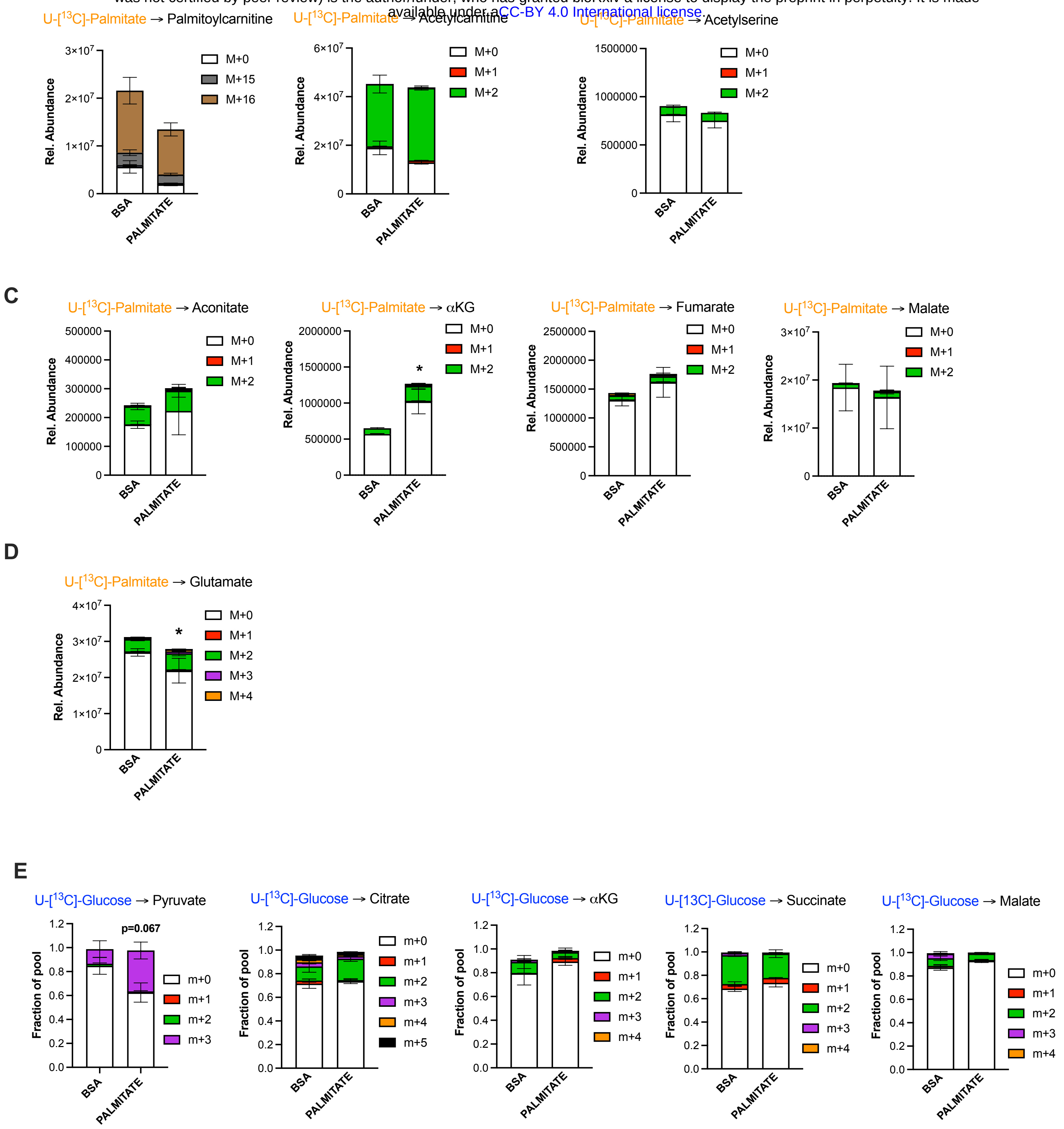

Supp_Figure_4

https://doi.org/10.1101/2023.04.03.535373 The copyright holder for this preprint (which
lay the preprint in perpetuity. It is made
CC-BY 4.0 International license

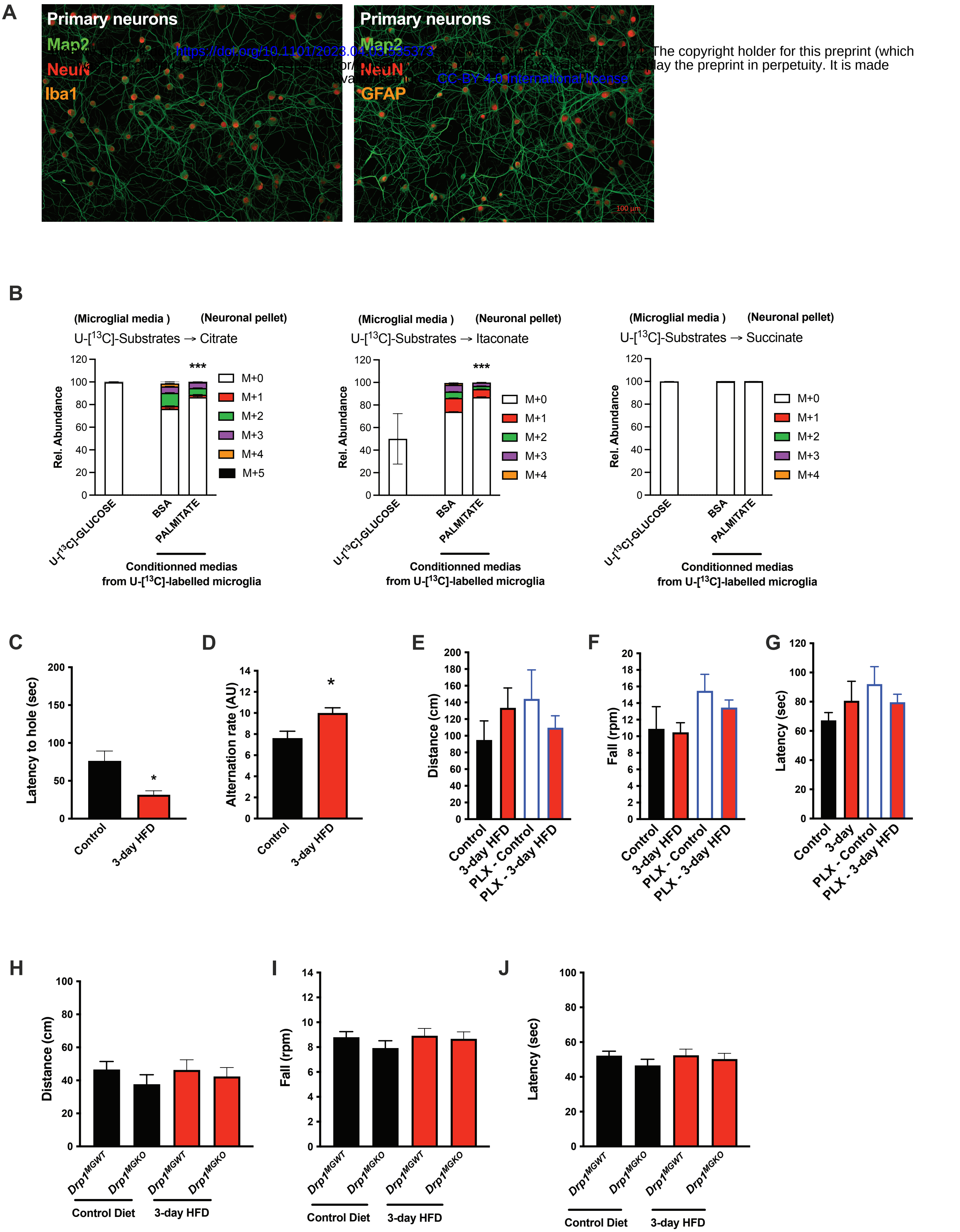

Supp_Figure_5

bioRxiv preprint doi: https://doi.org/10.1101/2023.04.03.535373; this version posted April 25, 2024. The copyright holder for this preprint (which was not certified by peer review) is the author/funder, who has granted bioRxiv a license to display the preprint in perpetuity. It is made available under aCC-BY 4.0 International license.

A

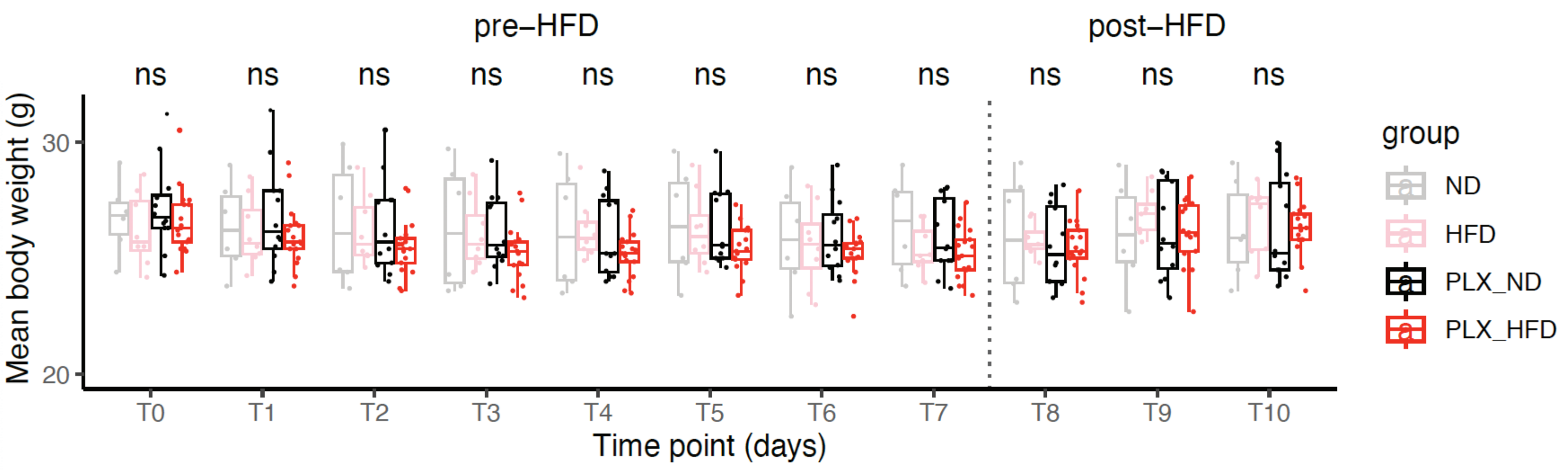

B

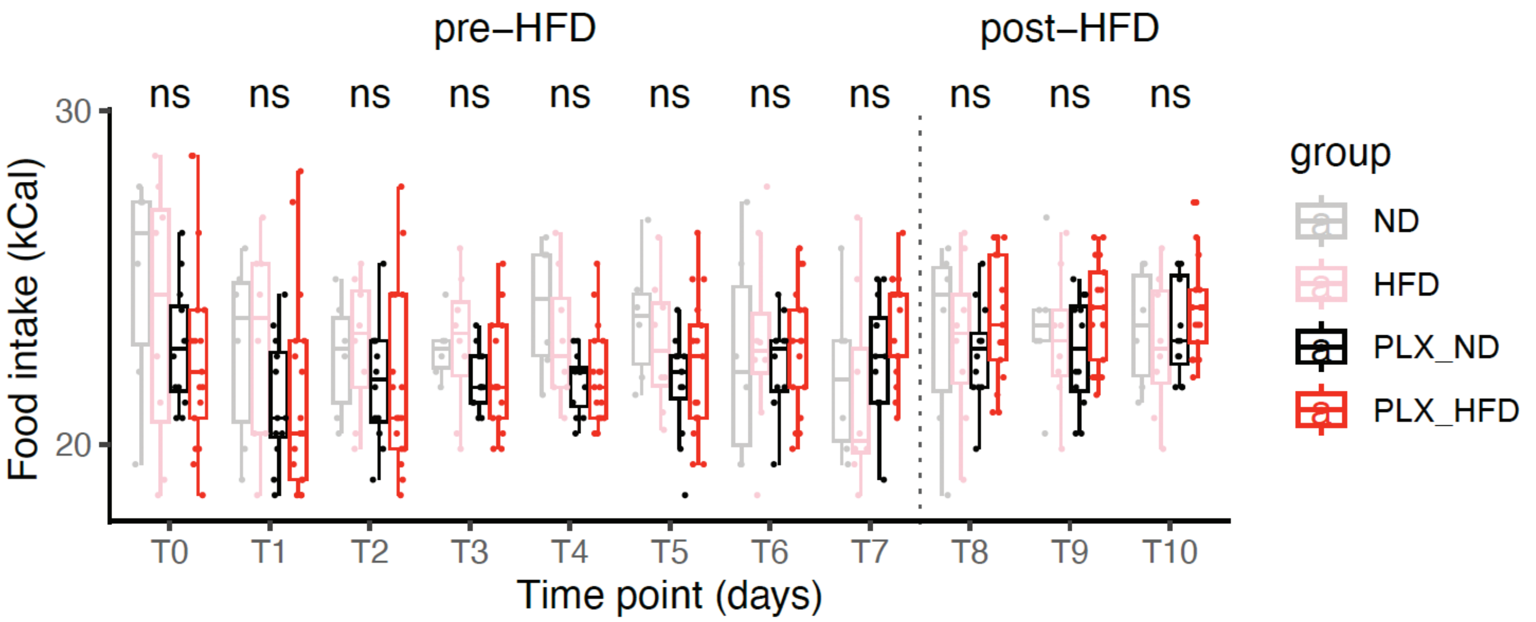

Supp_Figure_6

Table 2. Metabolites released in media from BSA- or palmitate (PA)-treated neurons

Characteristic	N	Overall, N = 12[1]	BSA, N = 6[1]	PA, N = 6[1]	p-value[2]	q-value[3]
b_DL.Lactic.Acid	12	7,768,093,932.0 (566,966,988.5)	7,315,358,105.8 (237,880,097.3)	8,220,829,758.2 (398,345,524.3)	0.002	0.064
b_D..Glutamine	12	92,514,878.8 (9,477,866.2)	99,910,567.8 (6,363,882.4)	85,119,189.7 (5,084,875.9)	0.002	0.064
a_Acetyl.L.carnitine	12	21,534,366.5 (5,136,659.2)	25,486,005.2 (4,313,399.0)	17,582,727.8 (1,401,821.0)	0.004	0.085
b_L.Serine	12	8,927,932.4 (1,999,447.4)	10,439,581.3 (1,620,380.1)	7,416,283.5 (827,833.2)	0.009	0.13
b_X4.Oxoproline	12	5,158,697.7 (2,093,379.7)	6,584,223.3 (1,496,000.9)	3,733,172.0 (1,589,279.7)	0.026	0.3
b_X3.Hydroxy.2.methyl.4.pyrone.tent.	12	112,324,855.5 (18,985,842.0)	122,558,080.8 (13,194,171.3)	102,091,630.2 (19,173,036.1)	0.065	0.6
a_Adipic.acid.tent.	12	463,554.3 (1,036,466.7)	0.0 (0.0)	927,108.7 (1,359,286.8)	0.074	0.6
a_D..Pyroglutamic.Acid	12	287,577,957.7 (32,240,216.1)	302,927,531.8 (25,661,286.0)	272,228,383.5 (32,600,385.7)	0.093	0.6
b_L.Arabinose.or.isomer.	12	427,996,608.9 (18,980,682.8)	437,653,974.3 (18,029,045.8)	418,339,243.5 (15,611,025.3)	0.093	0.6
b_L.Tyrosine	12	85,928,933.3 (296,055,668.3)	345,898.5 (293,093.1)	171,511,968.0 (418,627,435.0)	0.13	0.7
b_Sodium.lauryl.sulfate	12	22,598.3 (76,220.7)	0.0 (0.0)	45,196.5 (107,496.4)	0.2	0.7
b_Tridecanoic.acid	12	113,734.1 (302,240.1)	0.0 (0.0)	227,468.2 (412,217.0)	0.2	0.7
a_Leucine	12	27,980,174.9 (7,729,502.5)	30,862,636.3 (2,851,217.9)	25,097,713.5 (10,167,075.1)	0.2	0.7
b_Glutaric.acid.tent.	12	440,497,616.4 (19,061,443.4)	448,877,445.2 (22,010,672.2)	432,117,787.7 (12,097,256.5)	0.2	0.7
a_PEG.n5.tent.	12	344,189.2 (776,660.6)	4,541.3 (11,123.9)	683,837.0 (1,024,721.6)	0.2	0.7
a_L.Serine	12	390,966.4 (346,737.4)	527,905.0 (409,596.4)	254,027.8 (227,430.6)	0.2	0.7
b_D..Glucose.or.isomer.	12	5,354,625,351.9 (372,775,074.5)	5,219,148,234.7 (282,140,339.8)	5,490,102,469.2 (426,687,091.4)	0.2	0.7
b_AICA.ribonucleotide	12	64,660,392.8 (5,188,450.2)	63,405,196.7 (3,409,871.4)	65,915,588.8 (6,619,333.0)	0.2	0.7
b_neuraminic.acid.tent.	12	23,066,967.0 (2,864,562.7)	24,475,099.5 (3,412,576.8)	21,658,834.5 (1,283,781.1)	0.2	0.7
a_Betaine	12	3,612,697.5 (2,636,694.6)	2,762,143.5 (2,827,681.0)	4,463,251.5 (2,358,539.4)	0.3	>0.9
a_DL.Arginine	12	3,821,072.9 (7,222,808.1)	2,199,664.0 (5,388,054.4)	5,442,481.8 (8,912,410.1)	0.3	>0.9
a_L.Threonine	12	5,270,710.2 (5,478,082.2)	6,513,108.5 (5,492,782.8)	4,028,311.8 (5,669,696.2)	0.4	>0.9
a_Pantothenic.acid	12	22,359,057.0 (26,015,488.1)	28,020,428.8 (25,119,049.5)	16,697,685.2 (27,947,862.2)	0.4	>0.9
b_L.Leucine	12	6,646,608.2 (3,705,017.3)	7,399,027.0 (3,879,174.6)	5,894,189.3 (3,713,895.4)	0.4	>0.9
b_Crotonic.acid	12	16,108,825.9 (23,803,380.1)	23,947,637.5 (26,242,912.3)	8,270,014.3 (20,257,315.3)	0.4	>0.9
b_Succinic.acid	12	407,804.1 (869,276.7)	109,080.2 (267,190.7)	706,528.0 (1,173,394.8)	0.5	>0.9
a_L.Lysine	12	24,416,825.1 (28,518,776.0)	34,566,558.7 (36,434,829.3)	14,267,091.5 (14,648,122.2)	0.5	>0.9
b_Pyridoxal.tent.	12	777,482.7 (1,211,214.3)	598,171.7 (398,509.7)	956,793.7 (1,729,598.9)	0.5	>0.9
b_X2.C.Methyl.D.erythritol4.phosphate.tent.	12	643,051,857.5 (148,219,463.1)	606,454,245.0 (172,899,107.7)	679,649,470.0 (123,382,309.5)	0.5	>0.9
b_X2.Methylsuccinic.acid.tent.	12	287,186,644.6 (143,335,650.3)	264,074,195.0 (155,198,662.0)	310,299,094.2 (140,821,055.1)	0.5	>0.9
b_Pyruvic.acid.tent.	12	150,010,045.5 (9,715,420.7)	147,364,165.0 (6,350,912.8)	152,655,926.0 (12,268,697.7)	0.5	>0.9
a_Niacinamide	12	1,384,815.1 (2,023,883.3)	1,614,438.5 (2,373,808.4)	1,155,191.7 (1,802,752.7)	0.5	>0.9
a_X6.Methoxyquinoline.tent.	12	7,956,394.3 (26,119,894.0)	15,246,370.2 (37,028,395.4)	666,418.3 (1,516,716.5)	0.5	>0.9
a_L..Methionine	12	788,318.3 (1,316,035.4)	642,601.0 (747,341.7)	934,035.5 (1,789,080.3)	0.5	>0.9
b_X4.Hydroxyquinoline	12	2,294,626.8 (5,198,100.9)	3,743,521.2 (7,079,171.4)	845,732.5 (2,071,613.1)	0.6	>0.9
a_X2.2.6.6.Tetramethyl.4.piperidinol.tent.	12	3,349,971.7 (5,949,414.1)	4,474,480.0 (7,959,519.7)	2,225,463.3 (3,388,416.0)	0.6	>0.9
a_N.N.Diethylethanolamine.tent.	12	3,251,751.6 (3,370,326.9)	2,444,947.7 (2,152,767.5)	4,058,555.5 (4,335,133.4)	0.6	>0.9
b_L.Isoleucine	12	7,328,303.6 (7,854,185.2)	8,665,141.0 (7,496,666.0)	5,991,466.2 (8,673,233.1)	0.7	>0.9
a_X6.Methylquinoline.tent.	12	385.0 (699.5)	274.2 (671.6)	495.8 (772.0)	0.8	>0.9
a_Hypoxanthine	12	38,073.7 (83,225.3)	44,836.3 (109,826.1)	31,311.0 (55,377.5)	0.8	>0.9
b_p.Toluenesulfonic.acid.tent.	12	3,372,499.5 (6,572,551.8)	3,393,943.8 (5,259,090.6)	3,351,055.2 (8,208,375.3)	0.8	>0.9
b_Urocanic.acid.tent.	12	1,399.0 (2,534.9)	1,818.7 (2,821.3)	979.3 (2,398.9)	0.8	>0.9
a_Pyridoxal.tent.	12	1,928,431.0 (3,031,562.9)	2,203,965.7 (3,922,077.7)	1,652,896.3 (2,157,295.3)	0.8	>0.9
b_X3.Methyl.2.oxovaleric.acid	12	1,101,172.5 (3,039,252.9)	282,158.3 (278,456.3)	1,920,186.7 (4,316,724.3)	0.8	>0.9
b_L.Methionine	12	747,354.3 (822,900.9)	676,740.7 (845,132.3)	817,968.0 (873,813.0)	0.8	>0.9
b_L.Tryptophan	12	198,603.2 (296,935.2)	272,248.2 (407,584.4)	124,958.2 (121,792.6)	0.8	>0.9
a_Indole.3.acrylic.acid	12	2,553,283.9 (3,140,722.0)	2,083,026.7 (2,110,564.8)	3,023,541.2 (4,088,504.8)	0.8	>0.9
b_X4.Dodecylbenzenesulfonic.acid.tent.	12	10,280,591.5 (7,701,423.0)	8,121,727.2 (4,928,645.2)	12,439,455.8 (9,747,256.3)	0.8	>0.9
a_N.Acetylputrescine	12	386,960.3 (468,563.5)	391,056.2 (550,659.6)	382,864.3 (423,966.5)	0.9	>0.9
a_D..Proline	12	661,884.2 (1,149,335.2)	957,007.0 (1,564,926.8)	366,761.3 (498,103.3)	0.9	>0.9
a_X4.Aminonicotinic.acid.or.isomer.	12	126,303.5 (225,453.7)	94,034.3 (196,574.4)	158,572.7 (265,864.7)	>0.9	>0.9
a_Choline	12	174,331,021.6 (21,492,889.5)	171,722,892.2 (20,170,821.4)	176,939,151.0 (24,353,435.2)	>0.9	>0.9
b_Acetoacetic.acid	12	319,609,159.2 (14,397,923.5)	321,712,028.7 (13,338,036.2)	317,506,289.7 (16,356,781.0)	>0.9	>0.9
a_Isoleucine	12	30,943,808.3 (15,505,575.1)	29,880,732.5 (15,885,905.7)	32,006,884.2 (16,548,594.4)	>0.9	>0.9
b_L.Phenylalanine	12	9,511,810.9 (5,608,112.4)	10,045,803.3 (6,593,135.4)	8,977,818.5 (5,003,823.0)	>0.9	>0.9
a_Creatine	12	137,229.8 (326,719.4)	112,430.3 (275,396.9)	162,029.2 (396,888.8)	>0.9	>0.9
b_D..Fructose.or.isomer.	12	1,376,324,172.0 (124,013,739.0)	1,382,734,756.2 (112,787,783.2)	1,369,913,587.8 (144,965,454.4)	>0.9	>0.9
b_Folic.acid	12	3,351,599.5 (3,207,090.2)	4,052,769.5 (4,167,729.0)	2,650,429.5 (2,019,416.2)	>0.9	>0.9
b_Propylparaben.or.isomer.	12	255,003.3 (618,051.8)	190,425.0 (466,444.1)	319,581.7 (782,812.0)	>0.9	>0.9

bioRxiv preprint doi: https://doi.org/10.1101/2023.04.03.535373; this version posted April 25, 2024. The copyright holder for this preprint (which was not certified by peer review) is the author/funder, who has granted bioRxiv a license to display the preprint in perpetuity. It is made available under aCC-BY 4.0 International license.

[1] Mean (SD)

[2] Wilcoxon rank sum test; Wilcoxon rank sum exact test

[3] False discovery rate correction for multiple testing

bioRxiv preprint doi: https://doi.org/10.1101/2023.04.03.535373; this version posted April 25, 2024. The copyright holder for this preprint (which was not certified by peer review) is the author/funder, who has granted bioRxiv a license to display the preprint in perpetuity. It is made available under aCC-BY 4.0 International license.

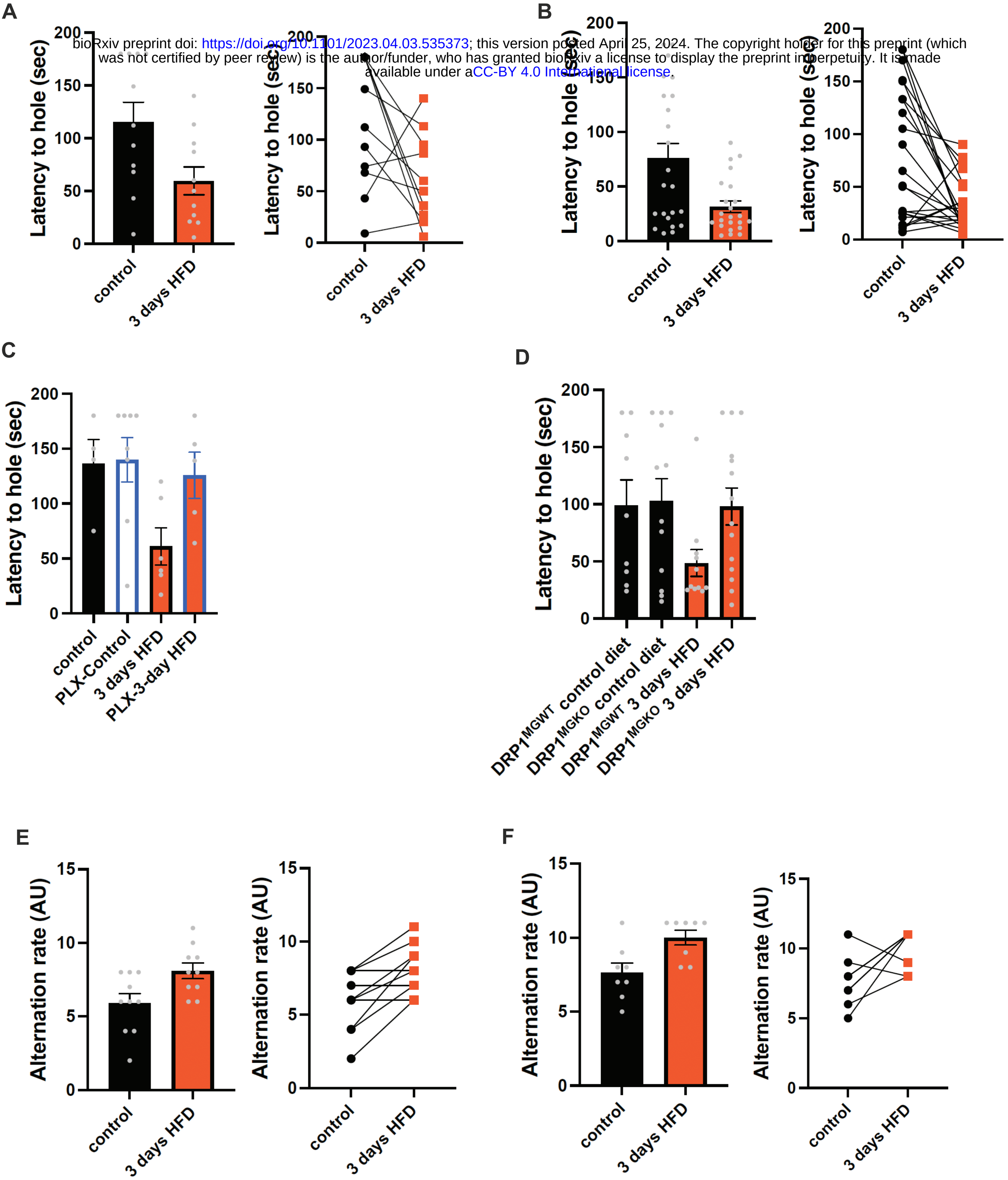

Supp_Figure_7

bioRxiv preprint doi: https://doi.org/10.1101/2023.04.03.535373; this version posted April 25, 2024. The copyright holder for this preprint (which was not certified by peer review) is the author/funder, who has granted bioRxiv a license to display the preprint in perpetuity. It is made available under aCC-BY 4.0 International license.

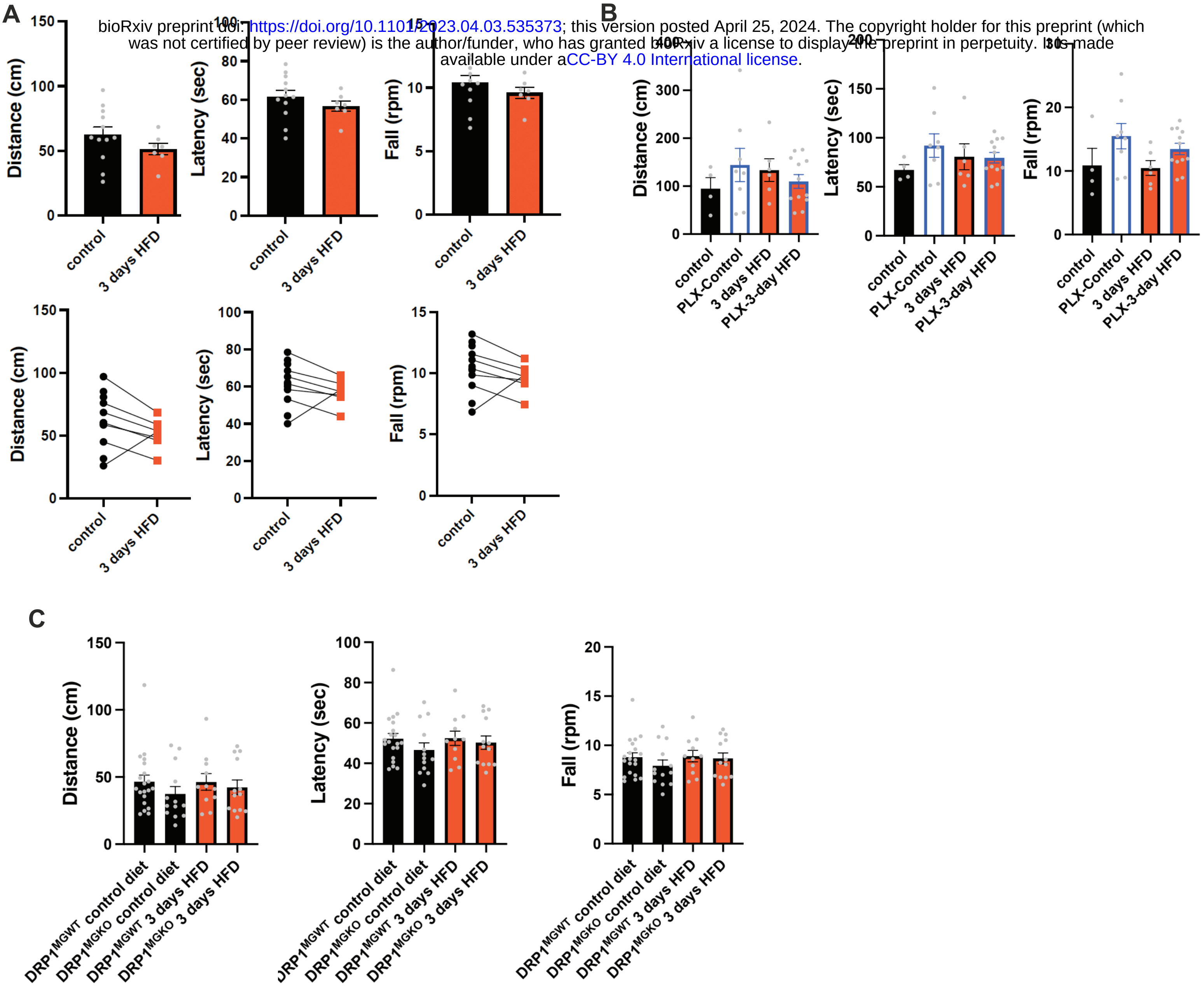

Supp_Figure_8

bioRxiv preprint doi: https://doi.org/10.1101/2023.04.03.535373; this version posted April 25, 2024. The copyright holder for this preprint (which was not certified by peer review) is the author/funder, who has granted bioRxiv a license to display the preprint in perpetuity. It is made available under aCC-BY 4.0 International license.

SUPPL FIGURE 1: Acute HFD-induced metabolic changes are microglia dependent

A. Graphs showing the Glucose Tolerance Test (OGTT) and the associated-insulin kinetic of C57Bl6/J male before keeping them with a control diet (Control) or before feeding them with high fat diet for 3 days (3-day HFD) (n=8).

B. Graph showing the Insulin Tolerance Test (ITT) of C57Bl6/J male before keeping them with a control diet (Control) or before feeding them with high fat diet for 3 days (3-day HFD) (n=5).

C. Graphs showing the Glucose Tolerance Test (OGTT) expressed in percentage from the basal glycemia of C57Bl6/J male fed with a control diet (Control) or fed with high fat diet for 3 days (3-day HFD) (n=8).

D. Graph showing the body weight evolution between C57Bl6/J male fed with control diet (Control) or fed with high fat diet for 3 days (3-day HFD), before and after the diet change. (n=7).

E. IBA1 immunostaining on hypothalamic slices from C57Bl6/J male fed with control diet (Control) or fed with high fat diet for 3 days (3-day HFD) and its quantification (n=10).

F. IBA1 and YFP immunostaining on hypothalamic slices from Cx3cr1creERT2-Rosa26YFP mice fed with control diet or 3 days high fat diet for 3 days (n=5).

G. sgRNAseq dataset from hypothalamic microglia cells harvested from C57bl6/J male mice fed with control diet (Control) and High Fat Diet for 3 days (HFD_3d) (n=5)

Data are presented as mean ±SEM. *p<0.05, **p<0.01, ***p<0.001 as determined by two-tailed Student's test and two-way ANOVA followed by Bonferroni post hoc.

SUPPL FIGURE 2: A rapid Microglial Mitochondria Response to high fat diet

A. IBA1 immunostaining of primary microglia cells treated with BSA (control) or Palmitate for 24hours.

B. Graph showing mRNA expression of microglial marker (TMEM119) or astrocytes marker (S100B) or macrophages marker (Arg1) in primary microglial culture challenged for 24hours with BSA or palmitate. Primary culture of astrocytes and macrophages were used as positive controls.

C. MitoSOX staining of primary microglial cells after being challenged for 24hours with BSA (control), Palmitate, Oleate or LPS (n=10) and the MitoSOX quantification graph.

D. Mitochondrial networks from primary microglia stained with Mitotraker green and stained with MitoSOX after being challenged for 2hours with BSA (control), Palmitate, Oleate or LPS (n=40) and the mitochondrial length as well as the MitoSOX quantification graphs

F. Interleukins concentrations (TNFalpha, IL-1beta, IL6) in the primary microglia cells media after being challenged for 24hours with BSA or palmitate (n=7)

Data are presented as mean ±SEM. *p<0.05, **p<0.01, ***p<0.001 as determined by two-tailed Student's test and two-way ANOVA followed by Bonferroni post hoc.

SUPPL FIGURE 3: aMMR is required for diet induced homeostatic rewiring *in vivo*

A Schematic depicting the timeline for the tamoxifen injection and the experiments performed on $Drp1^{MGWT}$ or $Drp1^{MGKO}$

B. Graph showing the body weight evolution among all the $Drp1^{MGKO}$ genotypes after the tamoxifen injection (n=6 to 11)

bioRxiv preprint doi: https://doi.org/10.1101/2023.04.03.535373; this version posted April 25, 2024. The copyright holder for this preprint (which was not certified by peer review) is the author/funder, who has granted bioRxiv a license to display the preprint in perpetuity. It is made available under aCC-BY 4.0 International license.

C. Graphs showing the fat mass and lean mass evolution among all the *Drp1*^{MGKO} genotypes after the tamoxifen injection (n=6 to 11)
D. Graphs showing the body weight before and after the 3 day HFD for the mice *Drp1*^{MGWT} and *Drp1*^{MGKO} (n=11 to 13)
Data are presented as mean ±SEM. *p<0.05, **p<0.01, ***p<0.001 as determined by two-tailed Student's test and two-way ANOVA followed by Bonferroni post hoc.

SUPPL FIGURE 4: Palmitate induces a novel microglial lactate/succinate/itaconate release pathway.
A ^{13}C-palmitate incorporation into palmytoilcarnitine and acetylcarnitine after 4 hours tracing experiment on primary microglia pretreated for 24hours with BSA or palmitate (n=3). The results are graphed in relative abundance.
B. ^{13}C-palmitate incorporation into acetyl-serine after 4 hours tracing experiment on primary microglia pretreated for 24hours with BSA or palmitate (n=3). The results are graphed in relative abundance.
C. ^{13}C-palmitate incorporation into aconitate, alpha-ketoglutarate, fumarate, malate after 4 hours tracing experiment on primary microglia pretreated for 24hours with BSA or palmitate (n=3). The results are graphed in relative abundance.
D. ^{13}C-palmitate incorporation into glutamate after 4 hours tracing experiment on primary microglia pretreated for 24hours with BSA or palmitate (n=3). The results are graphed in relative abundance.
E. ^{13}C-glucose incorporation into pyruvate, citrate, alpha-ketoglutarate, succinate, and malate after 6 hours tracing experiment on primary microglia pretreated for 24hours with BSA or palmitate (n=3). The results are graphed in pool size.
Data are presented as mean ±SEM. *p<0.05, **p<0.01, ***p<0.001 as determined by two-tailed Student's test and two-way ANOVA followed by Bonferroni post hoc.

SUPPL FIGURE 5: Acute HFD induces widespread MMR and rapid modulation of spatial and learning memory
A. Map2, NeuN, Iba1 and Map2, NeuN, GFAP immunostainings on primary neurons.
B. Primary microglial cell media was collected after the ^{13}C-glucose tracing (containing ^{13}C-metabolites released by microglia challenged with BSA or Palmitate) and incubated for 4 hours with primary neurons, the graph shows the ^{13}C-citrate, ^{13}C-itaconate and ^{13}C-succinate incorporation in the neurons in relative abondance (n=6).
C. Graph showing the latency during the Barnes Test from mice fed with normal diet (Control), or 3 days HFD (3-day HFD) (n=11). The test was performed in the MPI animals facility (Germany).
D. Graph showing the alternation during the T Maze Test from mice fed with normal diet (Control), or 3 days HFD (3-day HFD) (n=11). The test was performed in the MPI animals facility (Germany).
E. Graph showing the distance walked during the ROTAROD test from mice fed with normal diet (Control) (n=4), mice fed with 3 days HFD (3-day HFD) (n=6), mice depleted from their microglial cells with 1 week control diet complexed with PLX-5662 (PLX-Control) (n=8) or mice depleted

bioRxiv preprint doi: https://doi.org/10.1101/2023.04.03.535373; this version posted April 25, 2024. The copyright holder for this preprint (which was not certified by peer review) is the author/funder, who has granted bioRxiv a license to display the preprint in perpetuity. It is made available under aCC-BY 4.0 International license.

from their microglial cells with 1 week control diet complexed with PLX-5662 prior the 3 days HFD (PLX-3-day HFD) (n=12).
F. Graph showing the number of turns before the mice fall during the ROTAROD test from mice fed with normal diet (Control) (n=4), mice fed with 3 days HFD (3-day HFD) (n=6), mice depleted from their microglial cells with 1 week control diet complexed with PLX-5662 (PLX-Control) (n=8) or mice depleted from their microglial cells with 1 week control diet complexed with PLX-5662 prior the 3 days HFD (PLX-3-day HFD) (n=12).
G. Graph showing the latency during the ROTAROD test from mice fed with normal diet (Control) (n=4), mice fed with 3 days HFD (3-day HFD) (n=6), mice depleted from their microglial cells with 1 week control diet complexed with PLX-5662 (PLX-Control) (n=8) or mice depleted from their microglial cells with 1 week control diet complexed with PLX-5662 prior the 3 days HFD (PLX-3-day HFD) (n=12).
H. Graph showing the distance walked during the ROTAROD test from $Drp1^{MGWT}$ or $Drp1^{MGKO}$ mice fed with normal diet (Control diet), or 3 days HFD (3-day HFD) (n=11).
I. Graph showing the number of turns before the mice fall during the ROTAROD test from $Drp1^{MGWT}$ or $Drp1^{MGKO}$ mice fed with normal diet (Control diet), or 3 days HFD (3-day HFD) (n=11).
I. Graph showing the latency during the ROTAROD test from $Drp1^{MGWT}$ or $Drp1^{MGKO}$ mice fed with normal diet (Control diet), or 3 days HFD (3-day HFD) (n=11).
Data are presented as mean ±SEM. *p<0.05, **p<0.01, ***p<0.001 as determined by two-tailed Student's test and two-way ANOVA followed by Bonferroni post hoc.

SUPPL FIGURE 6:
A. Graph showing the body weight evolution between mice fed with normal diet or control diet complexed with PLX-5662 for 7days, prior the 3 days normal diet or 3days HFD. The 4 groups are the following :Control diet (ND)(n=6), 3days HFD (HFD) (n=8), 7day-PLX-5662 then control diet (PLX-ND) (n=12), 7day-PLX-5662 then 3-day HFD (PLX-HFD) (n=12).
B. Graph showing the food intake evolution between mice fed with normal diet or control diet complexed with PLX-5662 for 7days, prior the 3 days normal diet or 3days HFD. The 4 groups are the following :Control diet (ND)(n=6), 3days HFD (HFD) (n=8), 7day-PLX-5662 then control diet (PLX-ND) (n=12), 7day-PLX-5662 then 3-day HFD (PLX-HFD) (n=12).

SUPPL FIGURE 7:
A. Graphs showing the latency during the Barnes Test from **Fig 5D** with samples distribution and by using the paired comparison method.
B. Graphs showing the latency during the Barnes Test from **Suppl Fig S5C** with samples distribution and by using the paired comparison method.
C. Graphs showing the latency during the Barnes Test from **Fig 5J** with samples distribution and by using the paired comparison method.
D. Graphs showing the latency during the Barnes Test from **Fig 5k** with samples distribution and by using the paired comparison method.
E. Graphs showing the alternation during the T Maze Test from **Fig 5E** with samples distribution and by using the paired comparison method.
F. Graphs showing the alternation during the T Maze Test from **Suppl Fig S5D** with samples distribution and by using the paired comparison method.

bioRxiv preprint doi: https://doi.org/10.1101/2023.04.03.535373; this version posted April 25, 2024. The copyright holder for this preprint (which was not certified by peer review) is the author/funder, who has granted bioRxiv a license to display the preprint in perpetuity. It is made available under aCC-BY 4.0 International license.

SUPPL FIGURE 8:

A. Graphs showing the ROTAROD results (distance, latency, fall) from **Fig 5F-G-H** with samples distribution and by using the paired comparison method.

B. Graphs showing the ROTAROD results (distance, latency, fall) from **Suppl Fig S5E-F-G** with samples distribution and by using the paired comparison method.

C. Graphs showing the ROTAROD results (distance, latency, fall) from **Suppl Fig S5H-I-J** with samples distribution and by using the paired comparison method.
