# Supplementary material for: A rapid microglial metabolic response controls metabolism and improves memory
Source: bioRxiv. 2023 Apr 4:2023.04.03.535373. Preprint. [Version 1] doi: 10.1101/2023.04.03.535373 (PMC10103996; doi:10.1101/2023.04.03.535373)
Supplement: Supplementary file 1 [file NIHPP2023.04.03.535373v1-supplement-1.pdf]

bioRxiv preprint doi: https://doi.org/10.1101/2023.04.03.535373; this version posted April 7, 2023. The copyright holder for this preprint (which was not certified by peer review) is the author/funder, who has granted bioRxiv a license to display the preprint in perpetuity. It is made available under aCC-BY 4.0 International license.

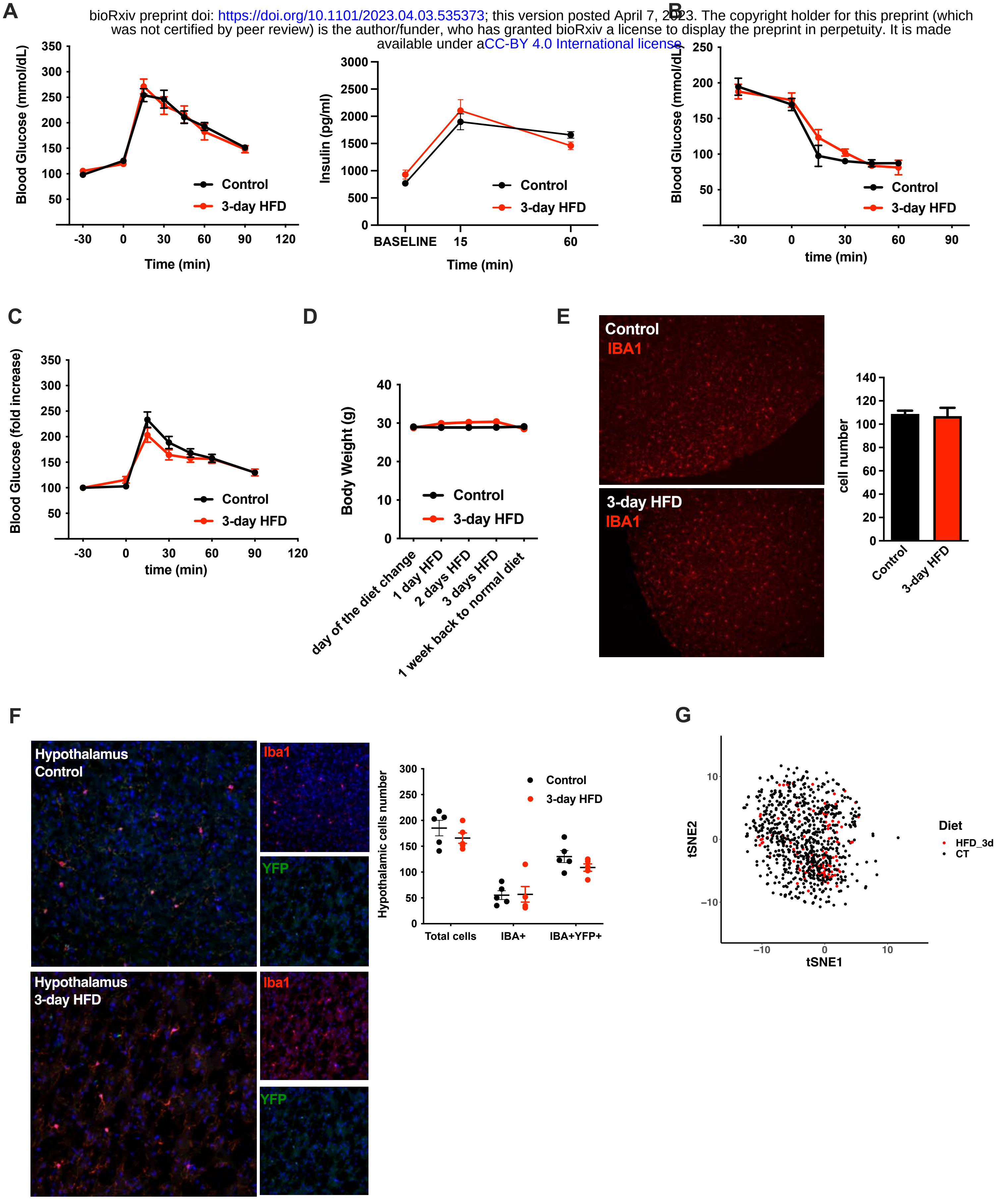

Supp_Figure_1

was not certified by peer review) is the author/funder, who has granted bioRxiv a license to display the preprint in perpetuity. It is made available under aCC-BY 4.0 International license.

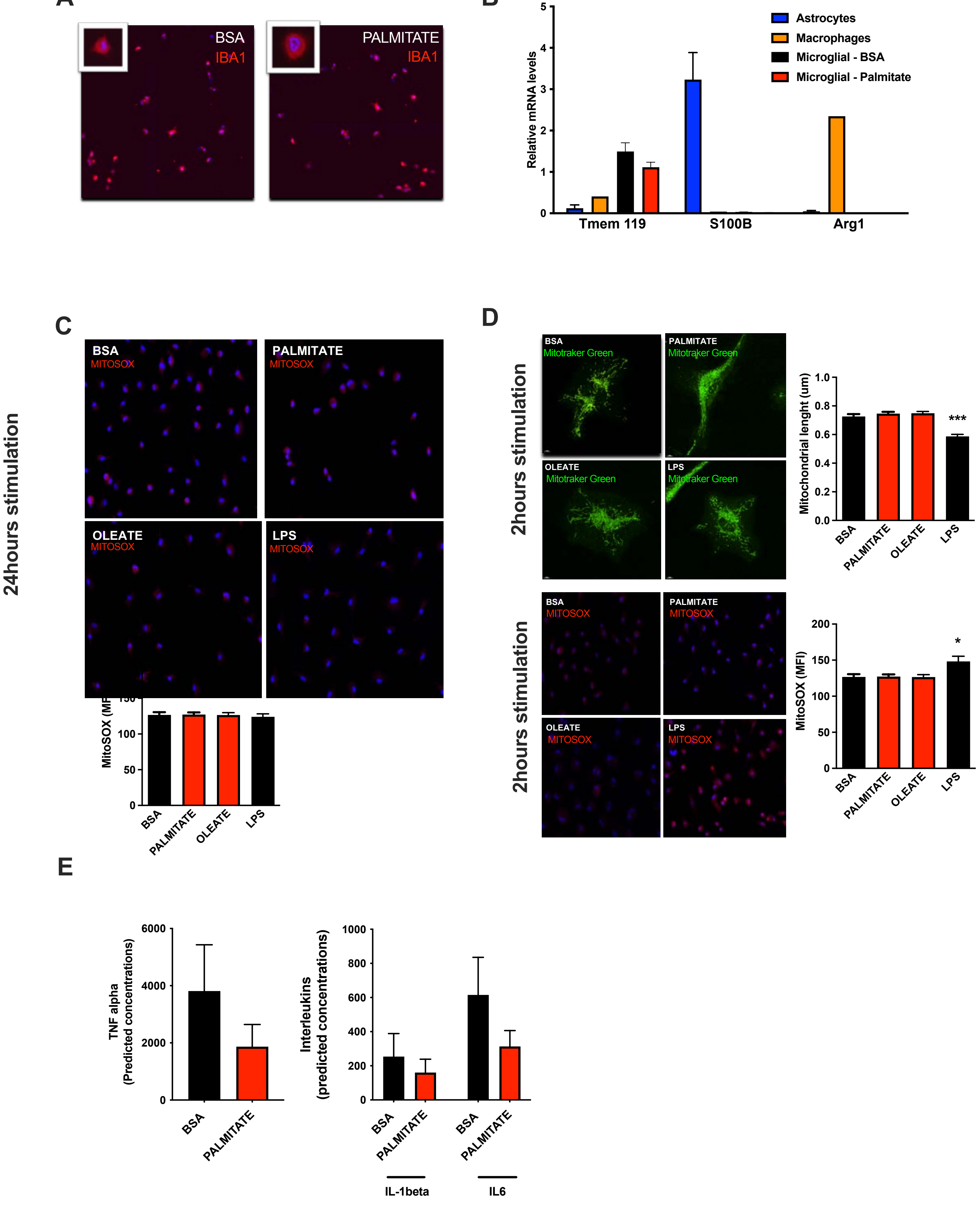

Supp_Figure_2

was not certified by peer review) is the author/funder, who has granted bioRxiv a license to display the preprint in perpetuity. It is made available under aCC-BY 4.0 International license.

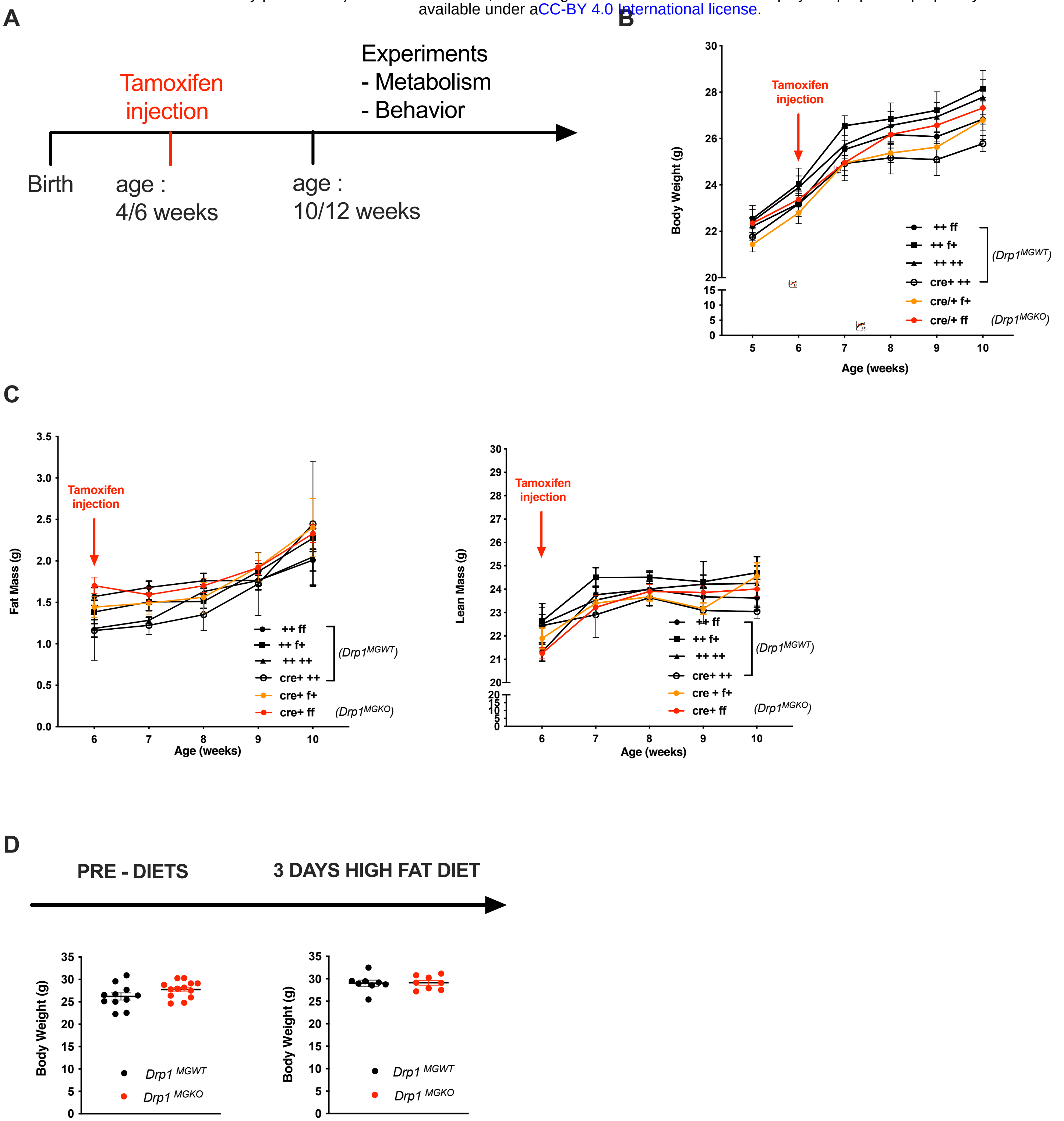

Supp_Figure_3

bioRxiv preprint doi: https://doi.org/10.1101/2023.04.03.535373; this version posted April 7, 2023. The copyright holder for this preprint (which was not certified by peer review) is the author/funder, who has granted bioRxiv a license to display the preprint in perpetuity. It is made available under aCC-BY 4.0 International license.

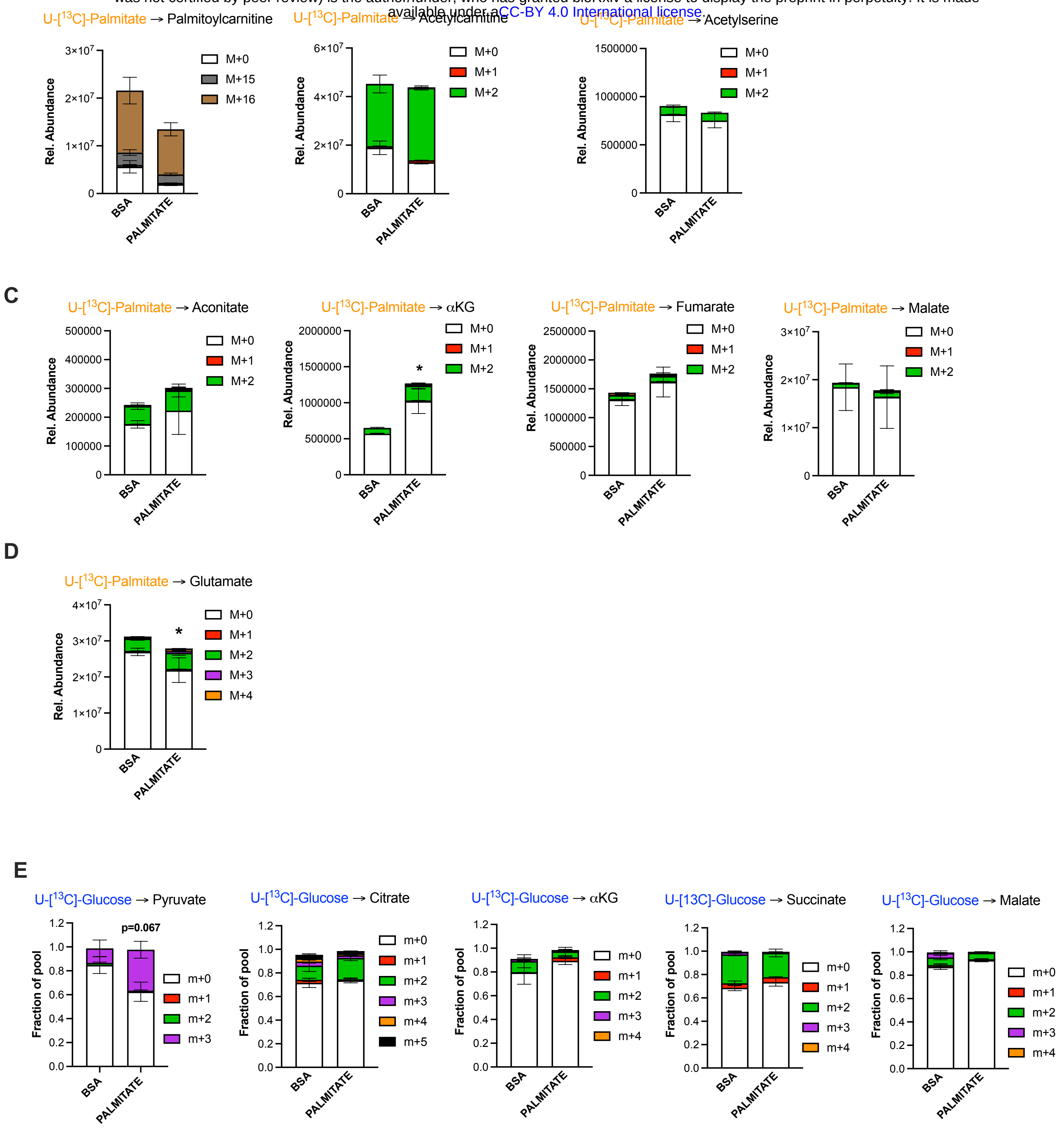

Supp_Figure_4

https://doi.org/10.1101/2023.04.03.535373 ...23. The copyright holder for this preprint (which ... display the preprint in perpetuity. It is made ... CC-BY 4.0 International license.

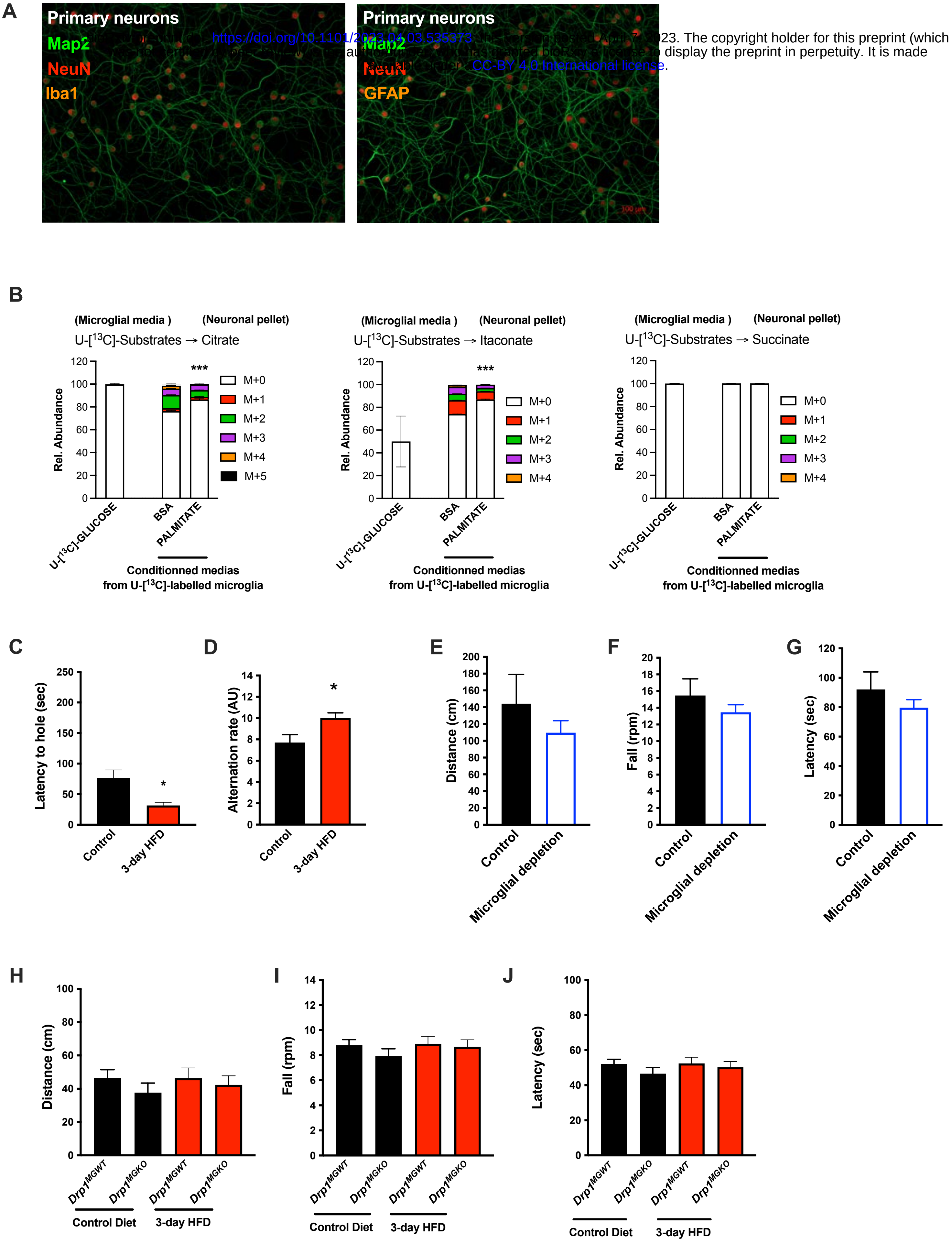

Supp_Figure_5

bioRxiv preprint doi: https://doi.org/10.1101/2023.04.03.535373; this version posted April 7, 2023. The copyright holder for this preprint (which was not certified by peer review) is the author/funder, who has granted bioRxiv a license to display the preprint in perpetuity. It is made available under aCC-BY 4.0 International license.

SUPPL FIGURE 1: Acute HFD-induced metabolic changes are microglia dependent

A. Graphs showing the Glucose Tolerance Test (OGTT) and the associated-insulin kinetic of C57Bl6/J male before keeping them with a control diet (Control) or before feeding them with high fat diet for 3 days (3-day HFD) (n=8).

B. Graph showing the Insulin Tolerance Test (ITT) of C57Bl6/J male before keeping them with a control diet (Control) or before feeding them with high fat diet for 3 days (3-day HFD) (n=5).

C. Graphs showing the Glucose Tolerance Test (OGTT) expressed in percentage from the basal glycemia of C57Bl6/J male fed with a control diet (Control) or fed with high fat diet for 3 days (3-day HFD) (n=8).

D. Graph showing the body weight evolution between C57Bl6/J male fed with control diet (Control) or fed with high fat diet for 3 days (3-day HFD), before and after the diet change. (n=7).

E. IBA1 immunostaining on hypothalamic slices from C57Bl6/J male fed with control diet (Control) or fed with high fat diet for 3 days (3-day HFD) and its quantification (n=10).

F. IBA1 and YFP immunostaining on hypothalamic slices from Cx3cr1creERT2-Rosa26YFP mice fed with control diet or 3 days high fat diet for 3 days (n=5).

G. sgRNAseq dataset from hypothalamic microglia cells harvested from C57bl6/J male mice fed with control diet (Control) and High Fat Diet for 3 days (HFD_3d) (n=5)

Data are presented as mean ±SEM. *p<0.05, **p<0.01, ***p<0.001 as determined by two-tailed Student's test and two-way ANOVA followed by Bonferroni post hoc.

SUPPL FIGURE 2: A rapid Microglial Mitochondria Response to high fat diet

A. IBA1 immunostaining of primary microglia cells treated with BSA (control) or Palmitate for 24hours.

B. Graph showing mRNA expression of microglial marker (TMEM119) or astrocytes marker (S100B) or macrophages marker (Arg1) in primary microglial culture challenged for 24hours with BSA or palmitate. Primary culture of astrocytes and macrophages were used as positive controls.

C. MitoSOX staining of primary microglial cells after being challenged for 24hours with BSA (control), Palmitate, Oleate or LPS (n=10) and the MitoSOX quantification graph.

D. Mitochondrial networks from primary microglia stained with Mitotraker green and stained with MitoSOX after being challenged for 2hours with BSA (control), Palmitate, Oleate or LPS (n=40) and the mitochondrial length as well as the MitoSOX quantification graphs

F. Interleukins concentrations (TNFalpha, IL-1beta, IL6) in the primary microglia cells media after being challenged for 24hours with BSA or palmitate (n=7)

Data are presented as mean ±SEM. *p<0.05, **p<0.01, ***p<0.001 as determined by two-tailed Student's test and two-way ANOVA followed by Bonferroni post hoc.

SUPPL FIGURE 3: aMMR is required for diet induced homeostatic rewiring *in vivo*

A Schematic depicting the timeline for the tamoxifen injection and the experiments performed on *Drp1*^{MGWT} or *Drp1*^{MGKO}

B. Graph showing the body weight evolution among all the *Drp1*^{MGKO} genotypes after the tamoxifen injection (n=6 to 11)

bioRxiv preprint doi: https://doi.org/10.1101/2023.04.03.535373; this version posted April 7, 2023. The copyright holder for this preprint (which was not certified by peer review) is the author/funder, who has granted bioRxiv a license to display the preprint in perpetuity. It is made available under aCC-BY 4.0 International license.

C. Graphs showing the fat mass and lean mass evolution among all the *Drp1^{MGKO}* genotypes after the tamoxifen injection (n=6 to 11)
D. Graphs showing the body weight before and after the 3 day HFD for the mice *Drp1^{MGWT}* and *Drp1^{MGKO}* (n=11 to 13)
Data are presented as mean ±SEM. *p<0.05, **p<0.01, ***p<0.001 as determined by two-tailed Student's test and two-way ANOVA followed by Bonferroni post hoc.

SUPPL FIGURE 4: Palmitate induces a novel microglial lactate/succinate/itaconate release pathway.
A ^{13}C-palmitate incorporation into palmytoilcarnitine and acetylcarnitine after 4 hours tracing experiment on primary microglia pretreated for 24hours with BSA or palmitate (n=3). The results are graphed in relative abundance.
B. ^{13}C-palmitate incorporation into acetyl-serine after 4 hours tracing experiment on primary microglia pretreated for 24hours with BSA or palmitate (n=3). The results are graphed in relative abundance.
C. ^{13}C-palmitate incorporation into aconitate, alpha-ketoglutarate, fumarate, malate after 4 hours tracing experiment on primary microglia pretreated for 24hours with BSA or palmitate (n=3). The results are graphed in relative abundance.
D. ^{13}C-palmitate incorporation into glutamate after 4 hours tracing experiment on primary microglia pretreated for 24hours with BSA or palmitate (n=3). The results are graphed in relative abundance.
E. ^{13}C-glucose incorporation into pyruvate, citrate, alpha-ketoglutarate, succinate, and malate after 6 hours tracing experiment on primary microglia pretreated for 24hours with BSA or palmitate (n=3). The results are graphed in pool size.
Data are presented as mean ±SEM. *p<0.05, **p<0.01, ***p<0.001 as determined by two-tailed Student's test and two-way ANOVA followed by Bonferroni post hoc.

SUPPL FIGURE 5: Acute HFD induces widespread MMR and rapid modulation of spatial and learning memory
A. Map2, NeuN, Iba1 and Map2, NeuN, GFAP immunostainings on primary neurons.
B. Primary microglial cell media was collected after the ^{13}C-glucose tracing (containing ^{13}C-metabolites released by microglia challenged with BSA or Palmitate) and incubated for 4 hours with primary neurons, the graph shows the ^{13}C-citrate, ^{13}C-itaconate and ^{13}C-succinate incorporation in the neurons in relative abondance (n=6).
C. Graph showing the latency during the Barnes Test from mice fed with normal diet (Control), or 3 days HFD (3-day HFD) (n=11). The test was performed in the MPI animals facility (Germany).
D. Graph showing the alternation during the T Maze Test from mice fed with normal diet (Control), or 3 days HFD (3-day HFD) (n=11). The test was performed in the MPI animals facility (Germany).
E. Graph showing the distance walked during the ROTAROD test from mice fed with normal diet (Control) or depleted from their microglia (n=8).

bioRxiv preprint doi: https://doi.org/10.1101/2023.04.03.535373; this version posted April 7, 2023. The copyright holder for this preprint (which was not certified by peer review) is the author/funder, who has granted bioRxiv a license to display the preprint in perpetuity. It is made available under aCC-BY 4.0 International license.

F. Graph showing the number of turns before the mice fall during the ROTAROD test from mice fed with normal diet (Control), (Control) or depleted from their microglia (n=8).
G. Graph showing the latency during the ROTAROD test from mice fed with normal diet (Control) or depleted from their microglia (n=8).
H. Graph showing the distance walked during the ROTAROD test from *Drp1*^{MGWT} or *Drp1*^{MGKO} mice fed with normal diet (Control diet), or 3 days HFD (3-day HFD) (n=11).
I. Graph showing the number of turns before the mice fall during the ROTAROD test from *Drp1*^{MGWT} or *Drp1*^{MGKO} mice fed with normal diet (Control diet), or 3 days HFD (3-day HFD) (n=11).
I. Graph showing the latency during the ROTAROD test from *Drp1*^{MGWT} or *Drp1*^{MGKO} mice fed with normal diet (Control diet), or 3 days HFD (3-day HFD) (n=11).
Data are presented as mean ±SEM. *p<0.05, **p<0.01, ***p<0.001 as determined by two-tailed Student's test and two-way ANOVA followed by Bonferroni post hoc.
